# Mechanisms of postoperative anorexia in surgical patients: a narrative review

**DOI:** 10.3389/fnins.2026.1710656

**Published:** 2026-03-02

**Authors:** Yanbo Sun, Zhichun Li, Ying Cai, Yunyun Cen, Yanli Li, Chengbin Li

**Affiliations:** 1 Department of Gastrointestinal Surgery, The Second Affiliated Hospital of Kunming Medical University, Kunming, China; 2 Center for Life Sciences, Yunnan Key Laboratory of Cell Metabolism and Diseases, School of Life Sciences, Yunnan University, Kunming, China; 3 Department of General Surgery, Caoxian People’s Hospital, Heze, Shandong, China; 4 Department of Anesthesiology, The Second Affiliated Hospital of Kunming Medical University, Kunming, China; 5 Department of Respiratory and Critical Care Medicine, Yan’an Hospital of Kunming City, Kunming, China; 6 Department of Hepatobiliary and Pancreatic Surgery, The Second Affiliated Hospital of Guangdong Medical University, Zhanjiang, Guangdong, China; 7 Zhanjiang Key Laboratory for Diagnosis and Repair of Digestive System Organ Injuries, Zhanjiang, Guangdong, China

**Keywords:** appetite, influencing factors, nutrition, perioperative, postoperative recovery

## Abstract

Postoperative anorexia is a highly prevalent condition among surgical patients, which exerting a profound impact on their recovery trajectories and nutritional status. The underlying mechanisms are complex and multifactorial, including neuroendocrine dysregulation, activation of inflammatory signaling pathways, and the interaction between psychological processes and pathological conditions. Emerging evidence underscores the significant role of altered hunger and satiety perception, cognitive modulation of food-related cues, and emotion-driven behavioral responses in the regulation of postoperative appetite. Despite these insights, there are currently no definitive targeted interventions available to effectively restore appetite in the postoperative setting. This narrative review summarizes recent advances in the understanding of appetite regulation, delineates key biological and psychosocial factors contributing to postoperative anorexia, and systematically synthesizes current clinical assessment approaches, and discusses emerging therapeutic strategies. By integrating insights from physiology, cognition, and affective science of postoperative anorexia, this narrative review seeks to provide a comprehensive understanding of the pathogenesis, assessment, and the current therapeutic strategies of postoperative anorexia.

## 1 Introduction

Postoperative anorexia is a prevalent challenge after surgery, significantly impacting patients’ recovery processes. It has been reported that up to 55% of patients continue to suffer from mild or severe postoperative anorexia within the first 2 weeks after colorectal surgery (Wennström et al., 2020). Moreover, patients undergoing joint replacement surgery may take as long as 4 weeks to regain their preoperative appetite (Prodger et al., 2016). These findings underscore not only the prevalence and persistence of postoperative anorexia but also the complex mechanisms of appetite regulation, in this context, the altered perception of hunger and satiety, along with the impaired cognitive integration of food-related stimuli, may collectively exacerbate nutritional deficits. Consequently, postoperative anorexia represents a critical barrier to optimal rehabilitation and timely recovery.

The mechanisms underlying postoperative anorexia remain unclear. As a result, there are currently no targeted clinical interventions to tackle its root causes (Fielding et al., 2023). In clinical practice, interventions such as nasogastric tube feeding, percutaneous gastrostomy, or parenteral nutrition are frequently employed to manage weight loss in patients who are unable to ingest sufficient nutrition orally. However, these approaches fail to address the emotional and psychological burden. In clinical practice, nor do they mitigate the anxiety and distress that accompany a reduced appetite frequently (Stratton, 2005).

Currently, postoperative nutritional support predominantly relies on passive treatment strategies, in which patients are supposed to accept nutritional interventions with limited active involvement. However, the compliance and responsiveness to such approaches are often limited. A central challenge in clinical nutritional care lies in the transition from passive acceptance to active engagement, thus promoting a more rapid recovery of appetite. Achieving this transformation necessitates a more profound understanding of the perceptual alterations in hunger and satiety, the cognitive processes that mold patients’ attitudes toward food, and the affective factors, such as anxiety or diminished motivation, which influence feeding behavior. Integrating these aspects into clinical practice may provide a more effective approach to improving postoperative nutritional outcomes.

Therefore, this narrative review synthesizes the mechanisms underlying postoperative anorexia, with a particular focus on altered perception of hunger and satiety, the disrupted cognitive regulation of food-related behaviors, as well as the affective and emotional factors that contribute to appetite loss. By incorporating biological, psychological, and behavioral perspectives, this narrative review seeks to provide a comprehensive and integrative understanding of the mechanisms underlying postoperative anorexia, systematically synthesizes current clinical assessment approaches, and deliberates on emerging therapeutic strategies.

This narrative review is structured to initially delineate the physiological regulation of appetite, subsequently investigate mechanisms specific to the postoperative state, and then conclude with a summary of assessment strategies and therapeutic implications relevant to clinical practice and future research. The literature for this narrative review was predominantly identified via searches on PubMed, using keywords such as “postoperative anorexia,” “postsurgical appetite loss,” “appetite regulation,” “gut-brain axis,” “neuroinflammation,” “cognitive regulation,” “affective processing,” and “surgical recovery.” Studies were selected based on their relevance to the integrative mechanistic framework of postoperative anorexia, which encompasses the interaction of several key systems after surgery, including neuroimmune activation, gut-brain signaling, hormonal changes, and the influence of mood and cognition on appetite following any type of general surgical operations. This narrative review focused on peer-reviewed English-language publications from the past two decades. However, given the limited body of literature on postoperative anorexia, seminal earlier studies were also incorporated where necessary to contextualize key mechanistic concepts.

## 2 Regulation of appetite under physiological conditions

**FIGURE 1:**
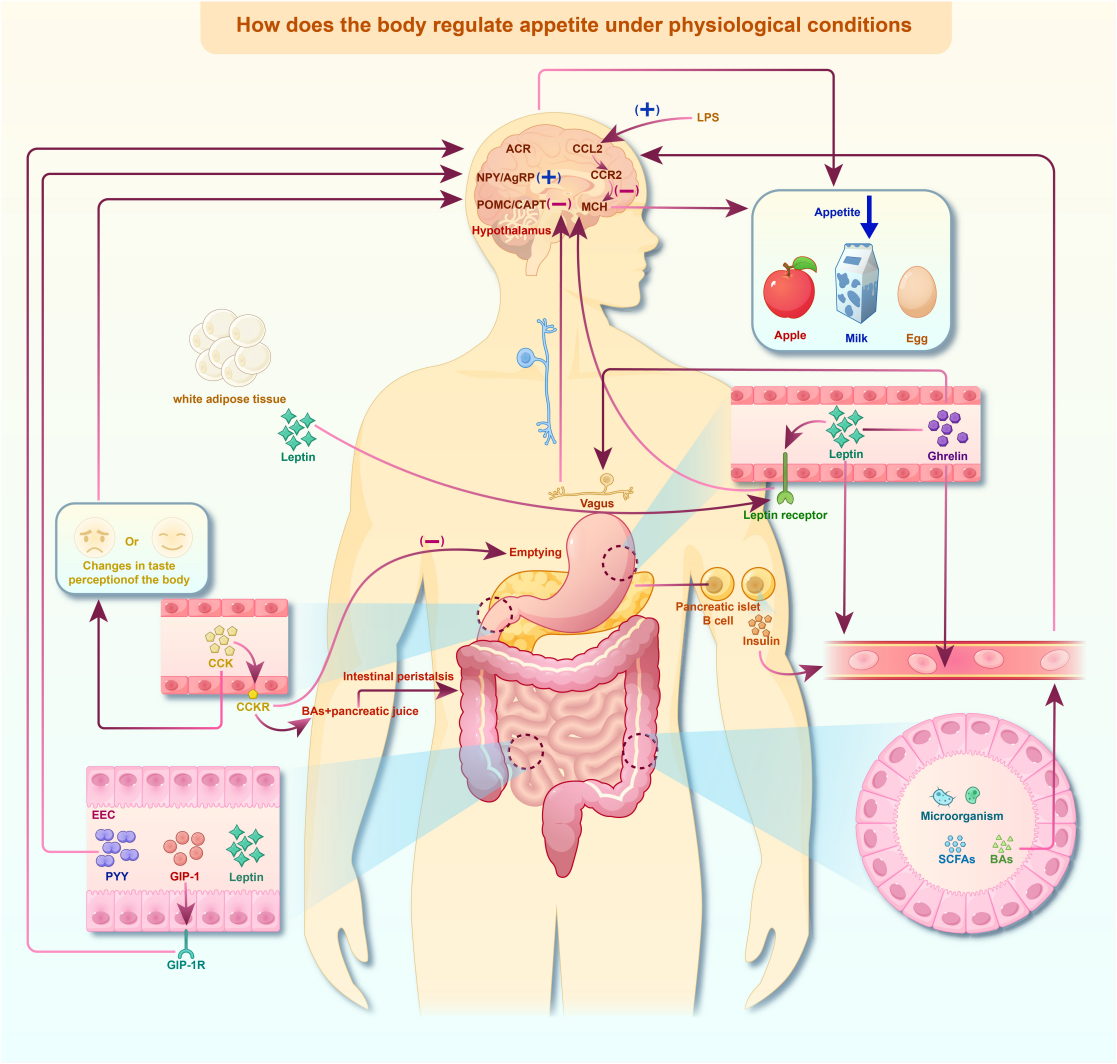
Regulation of appetite under physiological conditions. An overview of the integrated regulation of appetite under normal physiological conditions. This regulation mechanism involves central hypothalamic circuits, peripheral hormones, gastrointestinal signaling, vagal afferent pathways, and gut microbiota–derived metabolite. Arrows are used to indicate the direction of signal transmission, and the (+)/(–) symbols respectively denote the activation or suppression of downstream signaling or appetite - related responses. The pathway diagram was generated using Figdraw and adapted from the published literature.

The regulation of appetite involves complex interactions among diverse physiological systems, including the neurological, endocrine, and gastrointestinal systems, which rely on peripheral signals and mediators such as neuropeptides in the central nervous system (CNS) (Alonso et al., 2024). The hypothalamus acts as a central control hub for regulating appetite-stimulation responses (Perry and Wang, 2012), modulating the output of hunger signals. In addition to these physiological mechanisms, appetite regulation is substantially influenced by perceptual processes, the cognitive control of food-related cues, and affective and emotional states that mold eating motivation. Any disruptions in these interconnected domains, whether they are neural, cognitive, or affective, can cause abnormal fluctuations in appetite, resulting in significant clinical and health consequences (Han et al., 2021; Figure 1).

### 2.1 Central signaling pathways

A growing body of studies has shown that postoperative anorexia is not only driven by peripheral inflammation and gut hormone dysregulation but also stems from the functional remodeling of the central interoceptive network (Browning et al., 2017; Cosgrove et al., 2020; Martinou et al., 2022). Inflammatory, pain, and metabolic signals from the postoperative state enter the solitary tract nucleus and the parabrachial nucleus through the vagus afferent fibers and are further projected to key interoceptive regions, including the insula, anterior cingulate cortex, amygdala, and medial prefrontal cortex, which affect the subjective experience of hunger and satiety (Frank et al., 2013; Wright et al., 2016; Chiang et al., 2019; Al-Zubaidi et al., 2020). In the context of postoperative inflammation and autonomic nervous system imbalance, the insula’s sensitivity to hunger needs may decrease, while the responses of the anterior cingulate cortex and amygdala to discomfort, nausea, and negative body states may rise, resulting in the attenuation of the cortical representation of energy needs in the brain and the amplification of the representation of aversive signals (Chiang et al., 2019; Cosgrove et al., 2020; Rolls, 2023). Factors such as pain, opioid drugs, and postoperative ileus further exacerbate this “interoceptive misinterpretation,” causing patients’ lack of hunger and motivation to eat even when they are in a state of energy deficiency (Chen et al., 2021). Therefore, the impaired cortical interoceptive processing plays a crucial role in postoperative anorexia, which emphasizes the significance of understanding the postoperative anorexia behavior from the central cortical level.

#### 2.1.1 Central nervous system (CNS)

The hypothalamus plays a central role in regulating appetite and maintaining energy homeostasis through a complex network of neural circuits (Ester-Nacke et al., 2024). The “primary” group of neurons situated near the periventricular organs integrates circulating appetite-regulating signals, including the anabolic neuropeptides neuropeptide Y (NPY)/agouti-related peptide (AgRP) and pro-opiomelanocortin (POMC) generated in the arcuate nucleus (ARC) (Wang et al., 2024). NPY/AgRP neurons promote food intake, whereas POMC derived peptides, such as cocaine and amphetamine-regulated transcript (CART) induce satiety and suppress feeding through the melanocortin 4 receptor (MC4R) (Baldini and Phelan, 2019; Figure 1).

Beyond these canonical pathways, the hypothalamus is also involved in the processing of hunger and satiety signals and functionally interacts with limbic circuits related to emotion regulation (Ahima and Antwi, 2008; Campos et al., 2022; Goel et al., 2025). In the postoperative context, even though these central integrative pathways maintain their structural integrity, surgery - specific factors, such as inflammation, pain, stress, and altered interoceptive signaling, can significantly alter their functional integration (Gautron and Layé, 2010; Jagric, 2021; Zahid et al., 2025). This, in turn, reshapes the motivational and affective influences on feeding behavior and contributes to postoperative anorexia.

After integrating peripheral signals, ARC neurons project to “secondary” neurons in various brain regions, particularly within the hypothalamus, including the lateral hypothalamic area (LHA) (Li et al., 2023), ventromedial hypothalamic area (VMH), and paraventricular nucleus (PVN) (Webster et al., 2020), to regulate appetite (Schneeberger et al., 2014). These downstream circuits not only modulate feeding behavior at the physiological level but also interact with neural pathways involved in processing of hunger and satiety signals, as well as the cognitive processes that influence decision-making and the motivational aspects of food intake.

#### 2.1.2 Gastrointestinal hormones

Gastrointestinal hormones exert a significant influence on central appetite regulation through multiple mechanisms. Key hormones, including leptin (Endomba et al., 2020), cholecystokinin (CCK) (Crosby et al., 2018), peptide YY (PYY) (Loh et al., 2015), glucagon-like peptide-1 (GLP-1) (Zhang et al., 2022), and serotonin (5-HT) (Song et al., 2024) regulate appetite by binding to specific receptors in the hypothalamus. These hormones activate POMC neurons and suppressing NPY expression in AgRP neurons, thereby establishing the neural balance between hunger and satiety signals. In addition to these canonical pathways, gastrointestinal hormones also influence the perception of internal metabolic states and interact with affective circuits, linking peripheral energy signals to motivational and emotional factors driving feeding behavior.

#### 2.1.3 Gut microbiota and its metabolites

The gut microbiota and its metabolites, such as short-chain fatty acids (SCFAs) and bile acids (BAs), are of crucial significance in appetite regulation via the gut-brain axis (Wang et al., 2024). SCFAs can cross the blood-brain barrier (BBB) and directly modulate the CNS, thereby influencing energy balance and the perception of hunger and satiety. BAs can also reach the hypothalamus, where they regulate appetite by activating specific receptors like the Takeda G protein-coupled receptor 5 (TGR5), affecting NPY/AgRP neurons (Perino et al., 2021; Barbut et al., 2024). Furthermore, microbiota-derived signals interact with higher-order cognitive and emotional processes, integrating peripheral metabolic cues with affective states and motivational drives that shape feeding behavior.

In addition to these mechanisms, SCFAs influence appetite by modulating the secretion of gut hormones such as GLP-1, PYY and ghrelin. These hormones are pivotal in appetite regulation: SCFAs stimulate the release of GLP-1 and PYY, which exert anorexigenic effects, and potentially affect ghrelin levels through alterations in gut microbiota composition (Psichas et al., 2015; Byrne et al., 2016; Jiao et al., 2020; Zambrano et al., 2024). Moreover, recent studies have indicated that bacterial proteins can mimic host signaling molecules involved in appetite regulation. For instance, caseinolytic protease B (ClpB), a protein produced by Enterobacteriaceae, is structurally similar to the human alpha-melanocyte-stimulating hormone (α-MSH), a key peptide that suppresses appetite. By mimicking α-MSH, ClpB may interact with the melanocortin system to regulate food intake, thus establishing a more direct connection between the microbiota and appetite regulation (Tennoune et al., 2014; Dominique et al., 2019, 2021; Arnoriaga-Rodríguez et al., 2020; Molfino et al., 2020). Importantly, these microbiota-derived signals not only influence neural pathways but also impact the perception of hunger, engage in interactions with cognitive processes that govern food related decision-making, and modulate affective and emotional states related to eating behavior. This discovery provides a foundation for investigating microbiota-based therapies to regulate appetite and the treatment of disorders such as obesity and anorexia.

#### 2.1.4 Inflammation

Inflammation exerts a substantial impact on appetite regulation through intricate interactions with critical brain regions, particularly the ARC and LHA. In experimental models, for example, the administration of lipopolysaccharide (LPS) induces systemic inflammation and activate specific neuroimmune pathways, including the CCL2/CCR2 signaling cascade, which contributes to appetite suppression. Notably, LPS-induced inflammation has been shown to inhibit melanin-concentrating hormone (MCH) neurons in the hypothalamus, resulting in reduced food intake (Cazareth et al., 2014; Le Thuc et al., 2016). Inflammatory signals alter sensory and interoceptive perception, disrupt cognitive appraisal of food-related cues, and trigger negative affective and emotional states that further attenuate eating motivation. These findings underscore the role of inflammation in linking immune activation with neural circuits involved in appetite regulation, as well as behavioral processes that impact food intake.

Additionally, inflammation can influence the CNS not only through the bloodstream but also via the vagus nerve, which expresses receptors for inflammatory cytokines (Sofroniew, 2014; Freitas et al., 2022; Mukherjee et al., 2023). This vagal signaling mechanism enables peripheral inflammation to directly affect brain regions involved in appetite regulation (Dunn et al., 2006; Quintana et al., 2006; Browning et al., 2017). Beyond its physiological function, vagal signaling also contributes to the interoceptive perception of bodily states, modulates cognitive processing of food-related signals, and interacts with affective and emotional circuits that drive motivational aspects of feeding behavior. Thus, the vagus nerve acts as a key interface connects systemic inflammation to both neural and psychological dimensions of appetite regulation, offering valuable insights into how immune activation may reshape eating behavior.

Furthermore, muramyl dipeptide (MDP) and peptidoglycan, which are the structural components of bacterial cell walls, activate the immune system through pattern recognition receptors such as Toll-like receptor 2 (TLR2) and nucleotide-binding oligomerization domain 2 (NOD2) (Uehori et al., 2005; Schäffler et al., 2014). These receptors, capable of recognizing conserved microbial components, influence appetite by altering central inflammatory signaling pathways, thereby establishing a direct connection between gut microbiota and host feeding behavior (Denou et al., 2015). Such immune-mediated signaling can disrupt sensory and interoceptive perception, impede cognitive processing of food-related cues, and trigger affective and emotional responses that collectively influence eating motivation. In this manner, microbes and their metabolites act as critical mediators that bridge immune activation with both physiological regulatory mechanisms and higher order psychological processes, reinforcing the microbiota’s role in appetite regulation.

Further studies have demonstrated that inhibiting central melanocortin signaling using agents such as TCMCB07 can alleviate inflammation induced anorexia (Zhu et al., 2020; Axiak-Bechtel et al., 2023). Moreover, inflammatory signaling through nuclear factor kappa-light-chain-enhancer of activated B cells (NF-κβ) has been shown to regulate the transcription of POMC in the hypothalamus, revealing a distinct pathway through which inflammation influence appetite (Chen et al., 2024). Activation of NF-κβ can promote the production of anorexigenic peptides, thereby contributing to appetite suppression during inflammatory conditions (Tominaga et al., 2016; Desai et al., 2022). The inflammatory modulation of melanocortin pathways may disrupt sensory and interoceptive perception, impair cognitive appraisal of food-related stimuli, and evoke affective and emotional responses that further reduce eating motivation. Collectively, these findings highlight the multifaceted mechanisms by which inflammatory signaling remodels appetite regulation across physiological, cognitive, and affective domains.

Finally, a broader spectrum of inflammatory pathways has been associated with the modulation of feeding behavior. For instance, studies have revealed that proinflammatory cytokines such as TNF-α and IL-1β modulate appetite by acting on both on the brain and the peripheral tissues (Forte et al., 2020; Odoj et al., 2021). These cytokine-driven effects extend beyond metabolic regulation, influencing the sensory perception of bodily states, altering cognitive evaluation of food-related cues, and triggering affective and emotional responses that reduce eating motivation. Other studies further emphasize the complex interaction between inflammation and dietary behavior, suggesting that immune-driven changes in cognition and affective states may be as significant as direct metabolic alterations (Li et al., 2021).

### 2.2 Peripheral signaling pathways

The peripheral nervous system, particularly the vagus nerve, plays a crucial role in regulating food intake and energy homeostasis. Afferent fibers of the vagus nerve detect mechanical distension caused by gastric content (Müller et al., 2022), generating interoceptive signals that are transmitted to the CNS to modulate appetite perception and guide feeding behavior (Schwartz et al., 2000). In bariatric surgeries like Roux-en-Y gastric bypass (RYGB), although the vagus nerve pathway may remain intact, functional alterations post-surgery can disrupt its signaling capacity, thereby influencing appetite regulation (Hankir et al., 2017). These physiological changes extend beyond purely physiological regulation, intersecting with cognitive appraisal of satiety, emotional responses to eating, and broader psychological adaptations that collectively determine postoperative eating behavior.

### 2.3 Gastrointestinal hormone signaling pathways

The physiological mechanism underlying postoperative anorexia is closely associated with circulating hormones secreted by peripheral organs, such as ghrelin, leptin, GLP-1, CCK, insulin, and PYY. These hormones regulate appetite by modulating neural circuits in the brain (van Duinkerken et al., 2021; Costa et al., 2022). They shape sensory perception of satiety and fullness, influence cognitive processes involved in food related decision making, and interact with emotional states that can either reinforce or suppress feeding behavior.

#### 2.3.1 Ghrelin

Ghrelin, known as the hunger hormone, rises prior to meals and declines after food intake. It is predominantly synthesized in the stomach and stimulates appetite by activating its receptor, the growth hormone secretagogue receptor (GHSR). This activation is mediated by the internalization of the GHSR receptor, where hunger signals are transmitted to the brain via vagus nerve afferent neurons (Liu et al., 2021). Moreover, Ghrelin also modulates perceptual sensitivity to food cues, influences cognitive processes such as rewards anticipation and decision making, and engages affective circuits that connect appetite with motivation and mood.

#### 2.3.2 Leptin

Leptin, primarily secreted by white adipose tissue (WAT), is an important satiety hormone. It is also secreted in smaller amounts by the gastrointestinal tract and acts on the hypothalamus to suppress appetite and enhance energy expenditure (Park et al., 2020).

#### 2.3.3 GLP-1

Glucagon-like peptide-1 (GLP-1) plays a central role in regulating appetite and energy homeostasis. Released by intestinal L cells, GLP-1 acts on regions of the CNS involved in food intake and energy regulation by binding to the GLP-1 receptor (GLP-1R) (Singh et al., 2022).

#### 2.3.4 PYY

Peptide YY, secreted by intestinal L-type endocrine cells, primarily regulates appetite through modulation of the ARC region of the hypothalamus. There are two main circulating forms of PYY: PYY1-36 and PYY3-36, which have been shown to exert significant appetite-inhibitory effects in humans (Manning and Batterham, 2014; Igarashi et al., 2021).

#### 2.3.5 CCK

Cholecystokinin, synthesized in gastrointestinal tract cells, particularly in the duodenum and jejunum, influences appetite by modulating gastric motility and stimulates bile acid release (Steinert et al., 2017; Cai et al., 2024) and reduces ghrelin secretion, thereby promoting satiety (Herness et al., 2002).

#### 2.3.6 Insulin

Insulin plays a critical role in regulating glucose metabolism and contributes to satiety signaling by acting on POMC/CART and NPY/AgRP neurons in the hypothalamus, thereby reducing food intake. It functions synergistically with leptin to modulate appetite pathways, including the Adenosine 5′-monophosphate (AMP)-activated protein kinase (AMPK) signaling pathway (Kim et al., 2010; van Golen et al., 2013; Chruvattil et al., 2017).

## 3 Postoperative anorexia: mechanisms and consequences

**FIGURE 2:**
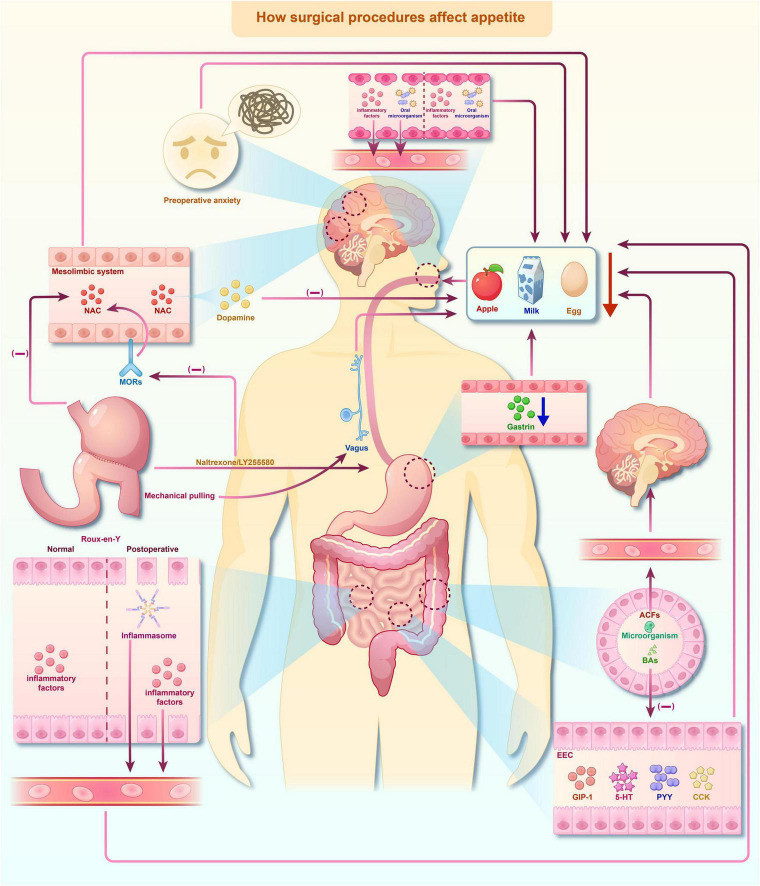
Impact of surgical procedures on appetite. Surgical procedures disrupt appetite regulation through the alteration of peripheral signals and central neural circuits, encompassing psychological, inflammatory, gastrointestinal, and neuroendocrine elements. Arrows are used to indicate the direction of signal transmission, and the (+)/(–) symbols respectively denote the activation or suppression of downstream signaling or appetite - related responses. The pathway diagram was generated using Figdraw and adapted from the published literature.

The decline in postoperative appetite is a multifaceted phenomenon influenced by a range of physiological and psychological factors. It has been observed in patients following various types of surgery, particularly abdominal surgery, bariatric surgery, and major surgeries requiring prolonged recovery. Multiple underlying mechanisms, including changes in gastrointestinal signaling, central neural pathways, inflammation, and hormonal fluctuations, contribute to this decline in appetite (Figure 2).

### 3.1 Gut-brain axis and postoperative anorexia

Postoperative anorexia is strongly associated with the comprehensive reconstruction of the intestinal end-signal environment (Sanmiguel et al., 2017; Hamamah et al., 2024). Following surgery, there is a decline in the diversity of the gut microbiota, a reduction in the production of key metabolites (such as SCFAs and bile acids), damage to the mucosal barrier, and local immune activation (Morrison and Preston, 2016; Medawar et al., 2021; Han et al., 2023; Song et al., 2025). These changes result in a substantial transformation of the chemical and mechanical signals transmitted from the intestine to the CNS. The weakened intestinal motility, delayed gastric emptying, and intestinal distension further modify the excitability of sensory nerves and vagal afferent fibers, changing the input signals from reflecting “energy demand” to a pattern more characterized by “fullness, discomfort, or defense” (Morrison and Preston, 2016; Medawar et al., 2021; Han et al., 2023; Song et al., 2025). In addition, postoperative sympathetic hyperactivity and decreased vagal tone can attenuate the crucial neural inputs that would otherwise signal energy deficiency (Cone et al., 2001; Morton et al., 2006). Overall, the composition and intensity of intestinal signals are systematically reshaped after surgery, causing the gut-brain axis information received by the CNS to inhibit food intake, thereby contributing to the occurrence of postoperative anorexia.

### 3.2 Inflammatory response and central appetite regulation circuit

Post-operative inflammation not only affects eating behavior through peripheral “disease signals” but also has the potential to directly act on the hypothalamic feeding regulation circuit. Surgical trauma results in a substantial elevation of pro-inflammatory cytokines, including IL-6, IL-1β, and TNF-α. These factors can enhance the permeability of the BBB under acute stress and enter the mediobasal hypothalamus (MBH) indirectly or directly by activating vascular endothelial cells, perivascular cells, and specialized brain barrier structures, thereby affecting the local neural activity of the ARC (Reyes and Sawchenko, 2002; Brown and Bradford, 2021; Galea, 2021; Yang et al., 2022). Previous studies have demonstrated that LPS and pro-inflammatory cytokines are capable of enhancing the excitability of POMC neurons to induced anorexia (Jang et al., 2010). NF-κβ suppresses food intake in mice by directly activating the Pomc promoter (Shi et al., 2013). TNF-α can suppress the discharge activity of AgRP/NPY neurons or modify their inhibitory synaptic inputs, resulting in a reduction in feeding-promoting signals and an elevation in anorectic signals (Hao et al., 2016; Chaves et al., 2020).

In addition to regulating neuronal excitability, inflammation is also capable of inducing changes in synaptic plasticity in the ARC region. Studies on animal models have revealed that acute systemic inflammation can reduce the dendritic spine density and excitatory synaptic inputs of AgRP neurons, while simultaneously increasing the excitatory afferents of POMC neurons, thereby further facilitating the over-activation of the melanocortin system (Nguyen and Dodd, 2024). This inflammation-driven circuit remodeling may endure beyond the acute post-operative stage, which accounts for the “prolonged recovery-type” appetite decline observed in some patients. Furthermore, in a systemic or metabolic inflammatory state, pro-inflammatory cytokines can facilitate the activation of microglia in the MBH, driving focal “micro-inflammation” and the NF-κβ cascade amplification reaction. This aberrant glial-neuronal signal can impair the normal responses of energy-sensing neurons (such as AgRP/POMC) to signals like hunger and leptin, consequently attenuating the compensatory eating behavior in the face of energy deficiency (Bhusal et al., 2021; Liu et al., 2025). These mechanisms, combined with postoperative pain and physical discomfort, directly suppress hunger and eating motivation independent emotional or cognitive factors, thus serving as an important central basis for postoperative anorexia.

### 3.3 Hormonal alteration and postoperative anorexia

Surgical procedures, particularly those involving the gastrointestinal tract, frequently result in significant alterations in circulating levels of key hormones that regulate appetite. For instance, bariatric surgery is associated with a marked reduction in ghrelin levels, which correlates strongly with decreased hunger. Conversely, satiety hormones, such as GLP-1 and PYY, may increase after such procedures, further decreasing appetite. These hormonal shifts are often compounded by changes in the gut microbiota composition, which may enhance the anorexic effect of postoperative states (Engel et al., 2023). Furthermore, evidence suggests that the interplay between these hormonal fluctuations and postsurgical inflammatory responses establishes a reinforcing feedback loop that sustains appetite reduction during recovery (Geisler and Hayes, 2023).

### 3.4 Psychological factors and postoperative anorexia

Psychological factors, including stress, anxiety, and depression, frequently contribute to postoperative anorexia. The psychological stress associated with surgery, compounded by pain and discomfort during recovery, can significantly reduce food intake. Moreover, Stress-related hormones, including cortisol and adrenaline, may further suppress appetite by affecting both the CNS and peripheral appetite signaling pathways (Goldstone et al., 2016). Post-surgical depression and anxiety are prevalent and can intensify anorexia. Studies have shown that patients with elevated levels of postoperative anxiety and depression tend to exhibit more severe anorexia and face greater challenges in regaining their normal eating behaviors compared to those with fewer psychological symptoms (Ullrich et al., 2013).

## 4 Assessment of postoperative anorexia

Accurate assessment of appetite is critical for identifying patients at risk of malnutrition and guiding early nutritional interventions (Abraham et al., 2019). Although no universal gold standard exists for evaluating postoperative anorexia, several validated questionnaires are widely used in clinical practice to quantify appetite and related symptoms (Table 1). These tools capture not only physiological dimensions of appetite but also subjective perceptual experiences of satiety and taste, cognitive evaluations about food intake, and affective or emotional responses that may shape eating behavior. The selection of the most appropriate instrument often depends on patient population, ease of application, and broader psychological context, underscoring the need for integrative assessment strategies that reflect both biological and psychosocial determinants of postoperative anorexia.

**TABLE 1:** Features of various tools used for evaluation of appetite status.

Assessment tool	Items	Assessment indicators	Intended population	Time needed for assessment	Reliability, validity, and other scale measures
CNAQ	8 clauses	Appetite; food intake	Adults	5 min	The sensitivity is 80.2%, and the specificity is 80.3% (5% decrease in body mass). The sensitivity is 82.4%, and the specificity is 81.9% (10% decrease in body weight)
SNAQ	4 clauses	Appetite; food intake	Adults	1 min	The sensitivity is 81.3%, and the specificity is 76.4% (5% decrease in body mass). The sensitivity is 88.2%, and the specificity is 83.5% (10% decrease in body weight)
DRAQ	10 clauses	Appetite; food intake; body mass	Patients with COPD	5 min	/
AHSPQ	29 clauses	Appetite	Older adults	15 min	Cronbach’s α coefficient is between 0.70 and 0.88
VAS	Between 5 and 10 clauses	Appetite	Older adults without cognitive impairment	5 min	/
FAACT	12 clauses	Anorexia; appetite	Patients with cancer	15 min	The sensitivity is 82%, and the specificity is 89%
CASQ	12 clauses	Appetite	Patients with cancer	15 min	The Cronbach’s α coefficient is 0.436, and the absolute value of factor loading for each item in the construct validity verification is between 0.481 and 0.893
A new model for nutritional status assessment	/	Physical function and capacity; health and somatic disorders; food and nutrition; cognitive, affective, and sensory function	Older adults	/	It is still in the research stage

### 4.1 The council on nutrition appetite questionnaire (CNAQ)

The CNAQ, developed by Wilson et al. (2005) is widely used due to its simplicity and effectiveness in predicting severe weight loss in individuals at risk for anorexia. The questionnaire yields a total score ranging from 8 to 40, with lower scores indicating worse appetite. Although primarily validated for use in older adults, it has also demonstrated applicability in younger patients. Its primary strengths lie in its brevity and ease of administration, making it well-suited for routine clinical settings.

### 4.2 The simplified nutritional appetite questionnaire (SNAQ)

The SNAQ is a shorter version of the CNAQ, with a total score range of 5–20 points (Wilson et al., 2005). A score below 14 indicates that patients at risk for severe weight loss. Due to its brevity and efficiency, the SNAQ is particularly valuable in settings requiring rapid assessment and is frequently used in busy clinical environments.

### 4.3 The disease-related appetite questionnaire (DRAQ)

Designed for patients with chronic obstructive pulmonary disease (COPD) (Nordén et al., 2015), the DRAQ focuses solely on appetite and provides a clear assessment of appetite status. With a total score range of 10–50 points, it serves as a valuable tool for individuals with chronic illnesses, though its applicability in postoperative settings may require further investigation. This instrument captures not only physiological aspects of appetite disturbances but also patients’ perceptual awareness of satiety, cognitive interpretations of eating behavior, and affective or psychological factors that influence their motivation to consume food. A recent study explored the application of DRAQ in hospitalized patients with gastrointestinal and liver disease and demonstrated its viability as a viable option for patients with prolonged recovery needs (Knudsen et al., 2015).

### 4.4 The appetite, hunger, sensory perception questionnaire (AHSPQ)

The AHSPQ, developed by de Jong et al. (1999) assesses appetite, hunger, taste, and smell perception with 29 items. Its broader scope makes it suitable for assessing sensory changes related to appetite loss. The AHSPQ is a validated, multidomain instrument that captures appetite and sensory-perception-related facets, with validation in geriatric settings, while it may be conceptually useful when sensory changes are clinically suspected after surgery, dedicated postoperative validation is limited, and postoperative sensory alterations are more directly documented by studies using other tools. However, its complexity limits its applicability in certain clinical environments.

### 4.5 Visual analogue scales (VAS)

The VAS is a psychometric instrument consisting of 7 items, each assessed on a 100-mm line. It is simple, versatile, and widely used in clinical research to evaluate subjective factors related to appetite, hunger, and satiety. By capturing patients’ perceptual experiences of bodily states and their cognitive evaluations of eating motivation, the VAS provides insights that extend beyond purely physiological measures. However, its reliance on subjective reporting may introduce variability across different populations and psychological contexts, particularly when emotional factors influence self-assessment. Recent perioperative and bariatric surgery investigations increasingly adopted the appetite visual analogue scale (VAS) as a patient-reported outcome, frequently combined with objective biomarkers (such as GLP-1, PYY, ghrelin or inflammatory markers), to triangulate postoperative appetite recovery (King et al., 2025).

### 4.6 Functional assessment of anorexia/cachexia therapy (FAACT)

The FAACT questionnaire measures the impact of anorexia on quality of life in cancer patients (Davis et al., 2009; Fávaro-Moreira et al., 2016). Though comprehensive, its length and focus on cancer-related anorexia may limit its practicality in specific postoperative patient groups. Consequently, its applicability may be restricted to specific patient subgroups. While the FAACT is well validated in oncology settings, its application in non-cancer postoperative patients requires further validation (Blauwhoff-Buskermolen et al., 2016; Zhou et al., 2017).

### 4.7 The cancer appetite and symptom questionnaire (CASQ)

The CASQ, developed by Halliday et al. (2012) is designed to predict weight loss in cancer patients based on appetite changes. While this instrument has proven effective in oncology contexts, its direct applicability to non-cancer postoperative populations may be limited. The CASQ is particularly valuable in identifying early weight loss among patients undergoing cancer treatment, as it integrates both physiological indicators and subjective perceptual changes in appetite. Moreover, it considers cognitive aspects of symptom interpretation and emotional or psychological states that often accompany disease-related anorexia. Emerging evidence suggests that the CASQ may also have potential in monitoring postoperative recovery in oncology patients, especially for individuals undergoing major surgical procedures, where both psychological vulnerability and affective responses may influence nutritional trajectories (Spexoto et al., 2016, 2018).

### 4.8 Other novel assessment tools

A novel model proposed by Engelheart and Brummer (2018) assesses multiple aspects of nutritional status in older adults, including physical function, somatic disorders, and cognitive function. While this model requires further validation before widespread adoption in clinical practice.

### 4.9 Recommendations and clinical application

Given the variety of available assessment tools, the selection of a specific assessment method should be guided by the patient’s clinical condition. For general postoperative application, the SNAQ is recommended due to its simplicity and proven effectiveness in identifying patients at risk of significant weight loss. For cancer patients or individuals with chronic diseases, the FAACT or CASQ may be more appropriate, as they are designed to evaluate the broader impact of anorexia on quality of life and symptom burden. In cases where sensory alterations in taste and smell are suspected to contribute to anorexia, the AHSPQ could provide valuable diagnostic insights. Ultimately, integrating these assessment tools with other clinical indicators, such as body weight trends, inflammatory markers, and patient-reported outcomes, will enhance the accuracy, comprehensiveness and effectiveness of postoperative nutritional assessment. However, further studies are needed to compare the efficacy of these tools across diverse patient populations, particularly in the context of postoperative recovery.

## 5 Treatment of postoperative anorexia

In contemporary clinical practice, nutritional support strategies, including nasogastric tube feeding, percutaneous gastrostomy, and parenteral nutrition continue to be the most frequently employed interventions for postoperative patients suffering from severe postoperative anorexia. These approaches are fundamentally supportive and compensatory, with the aim of guaranteeing sufficient caloric and nutrient intake when oral feeding proves inadequate. Significantly, despite variations in their administration routes and levels of invasiveness, they do not directly address the root mechanisms of appetite regulation or the motivation to eat.

Currently, there exists no standardized treatment protocol for postoperative anorexia. Nevertheless, in recent years, research has proposed several potential directions from the perspectives of drugs, neuromodulation, and the gut-brain axis mechanism. Regarding drugs, ghrelin receptor agonists (such as Anamorelin) can enhance hunger and eating motivation by activating hypothalamic NPY/AgRP neurons and the mesolimbic rewards pathway. Clinical trials have proven their effectiveness in improving body weight, lean body mass, and subjective appetite scores in patients with cancer cachexia (Naito et al., 2022; Taniguchi et al., 2023; Fujii et al., 2025).

In addition, vagus nerve stimulation (VNS), which includes both invasive and transcutaneous auricular vagus nerve stimulation (taVNS) shows promise for partially restoring postoperative gut-brain communication by enhancing vagal tone, regulating gastric motility, and attenuating the inflammatory response linked to postoperative ileus (Zhu et al., 2021; Huang et al., 2025). These interventions align with the central mechanisms highlighted in this narrative review, including “weakened eating motivation” and “abnormal interoceptive processing.” This underscores that postoperative anorexia is not only due to nutritional deficits but also involves complex neuro-endocrine-immune dysfunction.

Given that postoperative anorexia is frequently accompanied by low mood, anxiety, catastrophic interpretations, and negative biases toward visceral sensations, psychological and behavioral interventions hold great value. Cognitive-behavioral therapy, mindful eating training, guided sensory stimulation of food cues, and structured eating exposure programs have been demonstrated to enhance eating motivation, reduce eating-related anxiety, and reshape individuals’ subjective assessment of satiety and discomfort in patients with various eating disorders and metabolic diseases (Waller and Beard, 2024; Kurnik Mesarič et al., 2025). Meanwhile, gut microbiota-based interventions (such as butyrate supplementation, probiotics, and prebiotics) may improve appetite and alleviate eating-related discomfort by enhancing the production of SCFAs (especially butyrate), regulating gut hormones like GLP-1 and PYY, and improving mucosal immunity and reducing low-grade inflammation (Larraufie et al., 2018; Coppola et al., 2021; Yu et al., 2024; Hamari et al., 2025). Overall, a multi-dimensional management approach that combines pharmacological intervention, neuromodulation, behavioral therapy, and microbiota regulation is expected to provide a more promising theoretical framework and a comprehensive practical pathway for the treatment of postoperative anorexia.

## 6 Conclusions and future outlook

Postoperative anorexia is a widespread clinical symptom that profoundly impairs nutritional status, delays recovery, and adversely affects long-term prognosis (Huckleberry, 2004; Tanaka et al., 2021; Bouloubasi et al., 2024). The evidence synthesized in this narrative review indicates that postoperative anorexia results from coordinated neuroimmune activation, altered gut–brain signaling, dysregulated appetite-related hormones, and disrupted interoceptive and affective processing. Rather than representing a simple caloric deficit, it reflects a multifactorial remodeling of central and peripheral appetite regulation systems. Pharmacological strategies such as corticosteroids, ghrelin analogs, and cannabinoids have been investigated, yet their long-term safety and efficacy remain uncertain (Katagiri et al., 2006; Cheung and Mak, 2009; Palus et al., 2011; Bowles et al., 2015).

Current evidence remains heterogeneous and is frequently sourced from experimental models or limited clinical cohorts. There is an inconsistent application of validated appetite assessment tools, which restricts direct clinical translation. Therefore, future research ought to prioritize mechanism - driven investigations that target neuroinflammatory pathways, gut–brain signaling circuits, appetite - related hormonal axes, and vagal–interoceptive regulation. Longitudinal, biomarker - integrated studies will be crucial for translating mechanistic insights into precision interventions that can restore physiological appetite regulation. Ultimately, the progress of postoperative appetite management will rely on integrating these targeted strategies within perioperative care frameworks.
